# Asparagine prevents intestinal stem cell aging via the autophagy‐lysosomal pathway

**DOI:** 10.1111/acel.14423

**Published:** 2024-11-25

**Authors:** Ting Luo, Liusha Zhao, Chenxi Feng, Jinhua Yan, Yu Yuan, Haiyang Chen

**Affiliations:** ^1^ Center of Gerontology and Geriatrics and Laboratory of Stem Cell and Anti‐Aging Research, National Clinical Research Center for Geriatrics and State Key Laboratory of Respiratory Health and Multimorbidity, West China Hospital Sichuan University Chengdu Sichuan China

**Keywords:** aging, asparagine, autophagy, *Drosophila*, gut, intestinal stem cell

## Abstract

The age‐associated decline in intestinal stem cell (ISC) function is a key factor in intestinal aging in organisms, resulting in impaired intestinal function and increased susceptibility to age‐related diseases. Consequently, it is imperative to develop effective therapeutic strategies to prevent ISC aging and functional decline. In this study, we utilized an aging *Drosophila* model screening of amino acids and found that asparagine (Asn), a nonessential amino acid in vivo, exhibits its profound anti‐aging properties on ISCs. Asn inhibits the hyperproliferation of aging ISCs in *Drosophila*, maintains intestinal homeostasis, and extends the lifespan of aging flies. Complementarily, Asn promotes the growth and branching of elderly murine intestinal organoids, indicating its anti‐aging capacity to enhance ISC function. Mechanistic analyses have revealed that Asn exerts its effects via the activation of the autophagic signaling pathway. In summary, this study has preliminarily explored the potential supportive role of Asn in ameliorating intestinal aging, providing a foundation for further research into therapeutic interventions targeting age‐related intestinal dysfunction.

## Graphical abstract

Our study pioneers the exploration of asparagine (Asn) in the context of intestinal aging. We demonstrate that Asn administration mitigates intestinal stem cell (ISC) hyperproliferation and gut functional decline in aged Drosophila and young flies with ISC‐specific ASNS depletion. Furthermore, we show that Asn enhances ISC function during aging by activating autophagy and inhibiting MAPK activity.

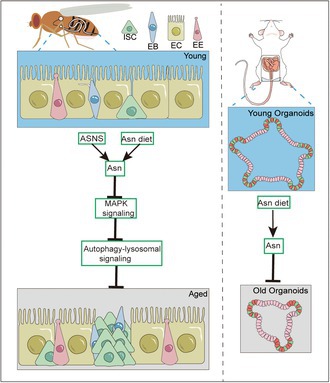

**Abbreviations**
Asn: asparagine
ASNS: Asparagine synthetase
CCR: copper cells region
Dl: Delta
EBs: enteroblasts
ECs: enterocytes
EEPs: enteroendocrine progenitor cells
EEs: enteroendocrine cells
Esg: Escargot
GFP: green fluorescent protein
ISC: intestinal stem cell
KEGG: Kyoto Encyclopedia of Genes and Genomes
MAPK: mitogen‐activated protein kinases
PCA: Principal component analysis
pERK: phosphorylated ERK
RNAi: RNA interference
RNA‐seq: RNA‐sequencing
Spd: Spermidine

## 1 INTRODUCTION

Stem cell aging is considered an important driver of organismal aging (Ren et al., 2017). The mammalian intestine, noted for its high cell turnover, is ideal for studying stem cell dynamics (Funk et al., 2020; Jasper, 2020), but aging causes intestinal stem cells (ISCs) to accumulate damage, reducing their quantity and functionality (B. Liu et al., 2022). Notably, age‐associated phenotypes can be ameliorated by the induction of stem cell rejuvenation in vivo (Rando & Chang, 2012). For instance, gut homeostasis, which is severely compromised with aging, can be improved by modulating ISC activity, subsequently extending lifespan (Rodriguez‐Fernandez et al., 2020). Consequently, developing interventions to counteract ISC aging and elucidating their underlying mechanisms are crucial for promoting intestinal health and mitigating the aging process (Herranz & Gil, 2018; Madeo et al., 2018; Rosen & Yarmush, 2023).

In healthy aging, amino acids play a pivotal role (Dato et al., 2018; Simpson et al., 2017). Research suggests that restricting the intake of specific amino acids, such as methionine and tryptophan, can decelerate the aging process (Simpson et al., 2017). Conversely, increasing certain amino acids like serine, threonine, and valine may accelerate cellular aging (Kim et al., 2014; Mirzaei et al., 2014). However, supplementation with particular amino acids such as glutamine (Bähler et al., 2013), leucine, cysteine, and their derivatives taurine and arginine (Riddle et al., 2016), could potentially alleviate aging outcomes. Furthermore, amino acid metabolism plays a critical role in the self‐renewal and differentiation processes of stem cells, with metabolites like proline, threonine, or methionine playing essential roles in embryonic stem cells (Kilberg et al., 2016); glutamine metabolism fuels the differentiation of mesenchymal stem cells (Ning et al., 2022). However, the roles of amino acids in ISC aging remain largely unexplored.

Asparagine (Asn), a nonessential amino acid synthesized in various organs and obtained through dietary intake, plays a crucial role in proliferating cells (Birsoy et al., 2015; Krall et al., 2021). Asn also serves as a precursor for the production of various nutrients such as glucose, proteins, lipids, and nucleotides (Yuan et al., 2024). Furthermore, clinical studies have revealed Asn levels decrease as aging in fasting serum samples from elderly cohorts (Castro et al., 2022; Pitkanen et al., 2003), a trend likewise observed in cerebrospinal fluid samples (F.‐C. Liu et al., 2023). Asparagine synthetase (ASNS), a catalytic enzyme for the synthesis of Asn, converts aspartate and glutamine into Asn and glutamate in an ATP‐dependent reaction (Lomelino et al., 2017; Yuan et al., 2024). Previous investigations into Asn and the *ASNS* gene have predominantly concentrated on elucidating their roles within the context of tumors (Halbrook et al., 2022; Jiang et al., 2021; Knott et al., 2018; Krall et al., 2021; H. Li et al., 2019). Nonetheless, research on the role of Asn and ASNS in aging and ISC functionality remains limited.

*Drosophila* melanogaster, with its advantageous genetic model organism traits (Fox et al., 2020; Hales et al., 2015), serves as an ideal model for studying ISC aging. During homeostasis, the fly midgut comprises ISCs and various differentiated cell types, closely mirroring the complexity of mammalian intestines (Jasper, 2020). The *Drosophila* midgut epithelium consists of a heterogeneous population of cells: ISCs are characterized by the expression of Delta (Dl), a ligand for the Notch receptor, and Escargot (Esg), a member of the SNAIL family of transcription factors; Progenitor cells include enteroblasts (EBs) and enteroendocrine progenitor cells (EEPs); Differentiated cell types encompass enterocytes (ECs) and enteroendocrine cells (EEs), each fulfilling specialized functions critical for maintaining intestinal homeostasis and function (Figure S1A). In aging flies, the gut is characterized by uncontrolled ISC proliferation, leading to disruption of intestinal homeostasis and function, making it an optimal model for screening the effects of small molecules on ISC aging (Choi et al., 2008; Funk et al., 2020; Lu et al., 2023; Rodriguez‐Fernandez et al., 2020; Su, 2019; Tan et al., 2023).

In this study, we employed the *Drosophila* model to screen multiple amino acids and discovered that Asn can enhance the function of aging *Drosophila* ISCs. Furthermore, Asn alleviated functional and barrier impairments in the aging *Drosophila* intestine. Knockdown of ASNS in ISCs resulted in phenotypes resembling intestinal aging. Our mechanistic investigations revealed that Asn inhibited intestinal hyperplasia and barrier disruption in the aging *Drosophila* intestine by activating the autophagy‐lysosomal signaling pathway. Additionally, we substantiated the conservative function of Asn in aging mammalian ISCs using an intestinal organoid model. These findings provide novel insights into the positive impact and mechanisms of Asn on aging ISCs and intestinal function, highlighting its scientific relevance in healthy aging and longevity management strategies.

## 2 RESULTS

### 2.1 Asn enhances the function of aged ISCs in *Drosophila*

**FIGURE 1:**
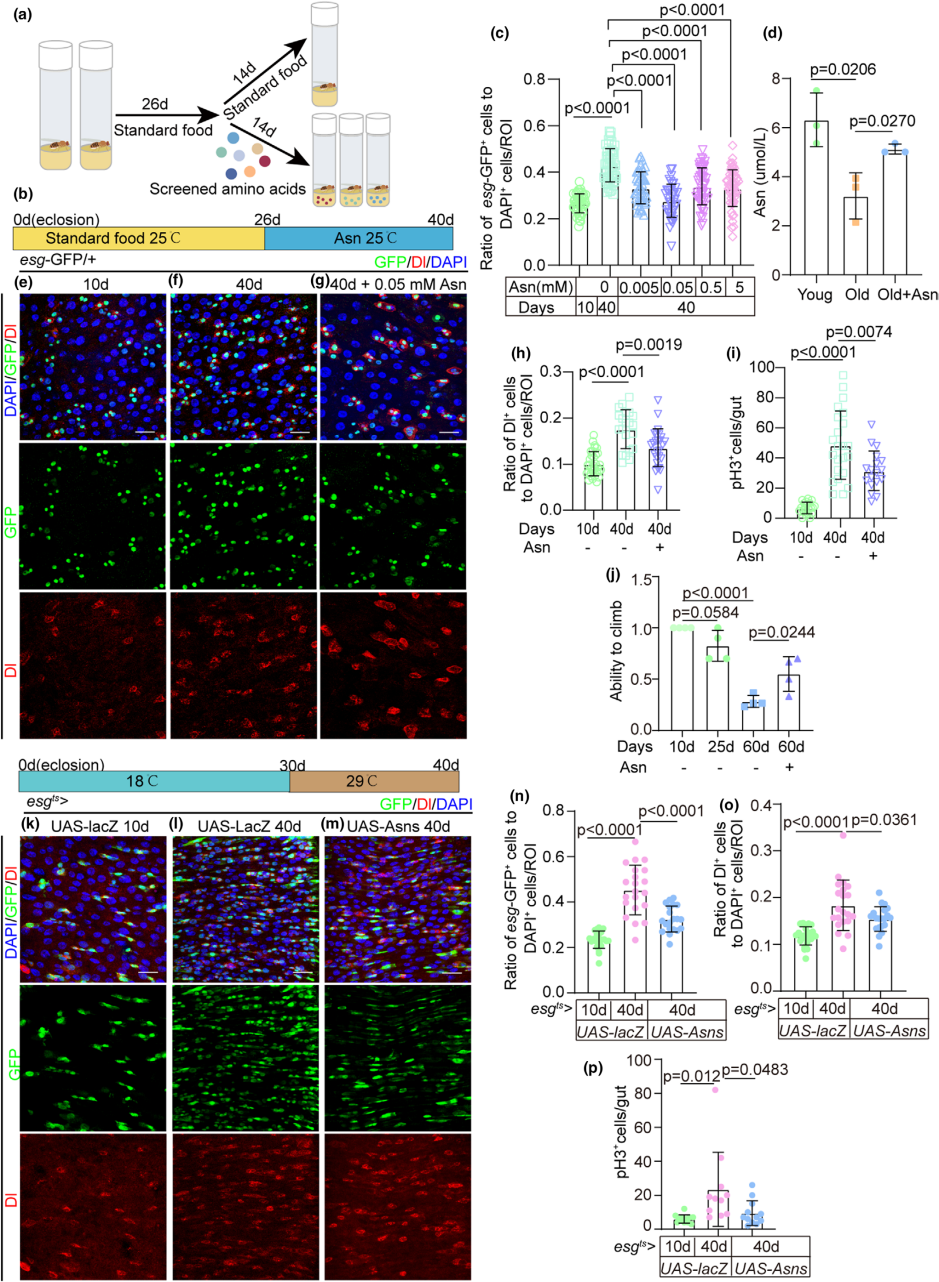
Asparagine (Asn) can enhance the function of aged ISCs in *Drosophila*. (a) The process of screening amino acids in the aged gut model of *Drosophila*. (b) Schematic diagram showing the process of feeding Asn to *Drosophila*. (c) The ratio of *esg‐GFP* ^+^ cells to DAPI^+^ cells per region of interest (ROI) in the posterior midguts of 10‐, and 40‐day‐old flies (*esg‐GFP/CyO*) without Asn supplementation and 40‐day‐old flies fed with four concentrations (0.005, 0.05, 0.5, and 5 mM) of Asn. *n*: Number of ROI counted. (*n* = 45, 48, 42, 38, 47,52, from left to right). (d) Intestinal Asn content was measured by the Asn kit in different groups of flies (the young group (control), the aging group, and the aging group supplemented with 5 μm Asn). (e–g) Representative immunofluorescence images of 10‐ (e), 40‐day‐old (f) posterior midguts without Asn supplementation, and 40‐day‐old posterior midguts with 0.05 mM Asn supplementation (g) stained with DAPI (blue; nuclei), GFP (green; ISCs and progenitor cells marker), and Dl (red; ISCs marker). The top panels represent the merged images, the middle panels represent *esg‐GFP*, and the bottom panels represent Dl. Scale bars represent 25 μm. (h) The ratio of Dl^+^ cells to DAPI^+^ cells per ROI in the posterior midguts of flies in experiments (e–g). *n*: Number of ROI counted. (*n* = 33, 21, 26, from left to right). (i) The number of pH 3^+^ cells in the whole guts of flies in experiments (Figure S1B–D). *n*: Number of guts counted. (*n* = 20, 23, 18, from left to right). (j) The ratio of good ability to climb in *Drosophila. n*: Number of tubes counted. (*n* = 4, 4, 4, 4, from left to right, 10 flies per tube). (k–m) Representative immunofluorescence images of 10‐ (k), 40‐day‐old (l) posterior midguts of *UAS‐lacZ* in *esg* ^+^ cells of flies, and 40‐day‐old posterior midguts of *UAS‐Asns* in *esg* ^+^ cells of flies (m) stained with DAPI (blue; nuclei), and pH 3 (red; ISC marker). Scale bars represent 25 μm. (n) The ratio of *esg‐GFP* ^+^ cells to DAPI^+^ cells per ROI in the posterior midguts of flies in experiments (k–m). *n*: Number of ROI counted. (*n* = 24, 21, 20, from left to right). (o) The ratio of Dl^+^ cells to DAPI^+^ cells per ROI in the posterior midguts of flies in experiments (k–m). *n*: Number of ROI counted. (*n* = 24, 21, 20, from left to right). (p) The number of pH 3^+^ cells in the whole guts of flies in experiments (k–m). *n*: Number of guts counted. (*n* = 12, 11, 12, from left to right). Scale bars represent 25 μm (e–g, k–m). Error bars represent standard deviations (SDs). Student's *t*‐tests and specific *p* values are indicated on the graphs.

Considering the unclear role of amino acids in ISC aging, we utilized a *Drosophila* model to screen efficient amino acids (Figure 1a). To assess the ISC function by quantifying its number, we used a reporter line (*esg*‐GFP/CyO) expressing green fluorescent protein (GFP), representing the *esg* gene. After our initial screening, Asn emerged as a potent inhibitor of *esg* ^+^ cell accumulation in the guts of aged *Drosophila*. We administered Asn to flies at the midlife stage (26 days) and performed dissections at an aged stage (40 days) for analysis (Figure 1b). Among the four tested concentrations (0.005, 0.05, 0.5, and 5 mM), 0.05 mM Asn demonstrated the most optimal effect (Figure 1c). Furthermore, we measured the Asn levels in the intestines of *Drosophila* from different groups using the Asn assay kit. Our results indicate that Asn levels in the intestines of aging flies are reduced compared to the young group. However, aging flies that were fed Asn exhibited elevated levels of Asn compared to those in the natural aging group (Figure 1d).

In young *Drosophila*, ISCs play a crucial role in maintaining intestinal homeostasis by proliferation and differentiation (Figure 1e). However, in aged *Drosophila*, the rate of ISC proliferation dramatically increases, leading to a substantial rise in the numbers of ISCs and progenitor cells (Figure 1f) (Choi et al., 2008; Cui et al., 2019; Rodriguez‐Fernandez et al., 2020). This hyperproliferation embodied a marked accumulation of *esg* ^+^ cells (which include ISCs and EBs) and Phospho‐Histone 3‐positive (pH 3^+^) cells (pH 3 specifically stains dividing ISCs) in aged *Drosophila* (Choi et al., 2008; Cui et al., 2019; Rodriguez‐Fernandez et al., 2020). The results indicated a significant reduction in the number of *esg* ^+^ cells in aged flies (40 days) fed with Asn compared to those without Asn (Figure 1f,g). Further quantification of Dl^+^ cells (a marker for ISC) and pH 3^+^ cells (a marker for proliferating cells) revealed that the numbers of Dl^+^ (Figure 1e–h) and pH 3^+^ (Figure 1i, S1B–D) cells in aged *Drosophila* treated with 0.05 mM Asn were notably less than those in the control group. These findings suggest that Asn supplementation prevents ISC overproliferation and subsequent intestinal hyperplasia in aging flies. Moreover, aging flies supplemented with Asn exhibit better climbing ability (which decreases with aging) (Liang et al., 2023; Rhodenizer et al., 2008; Wilson et al., 2020) compared to those that did not receive Asn supplementation (Figure 1j).

Moreover, we conducted experiments on the overexpression of ASNS in aging flies, and the results indicate that during aging, overexpression of ASNS can reduce the overproliferation of aging‐related ISCs, like the effects observed with the addition of Asn (Figure 1k–p).

### 2.2 The reduced Asn synthesis in the ISCs leads to the accumulation of ISCs and Asn extends the lifespan in *Drosophila*

**FIGURE 2:**
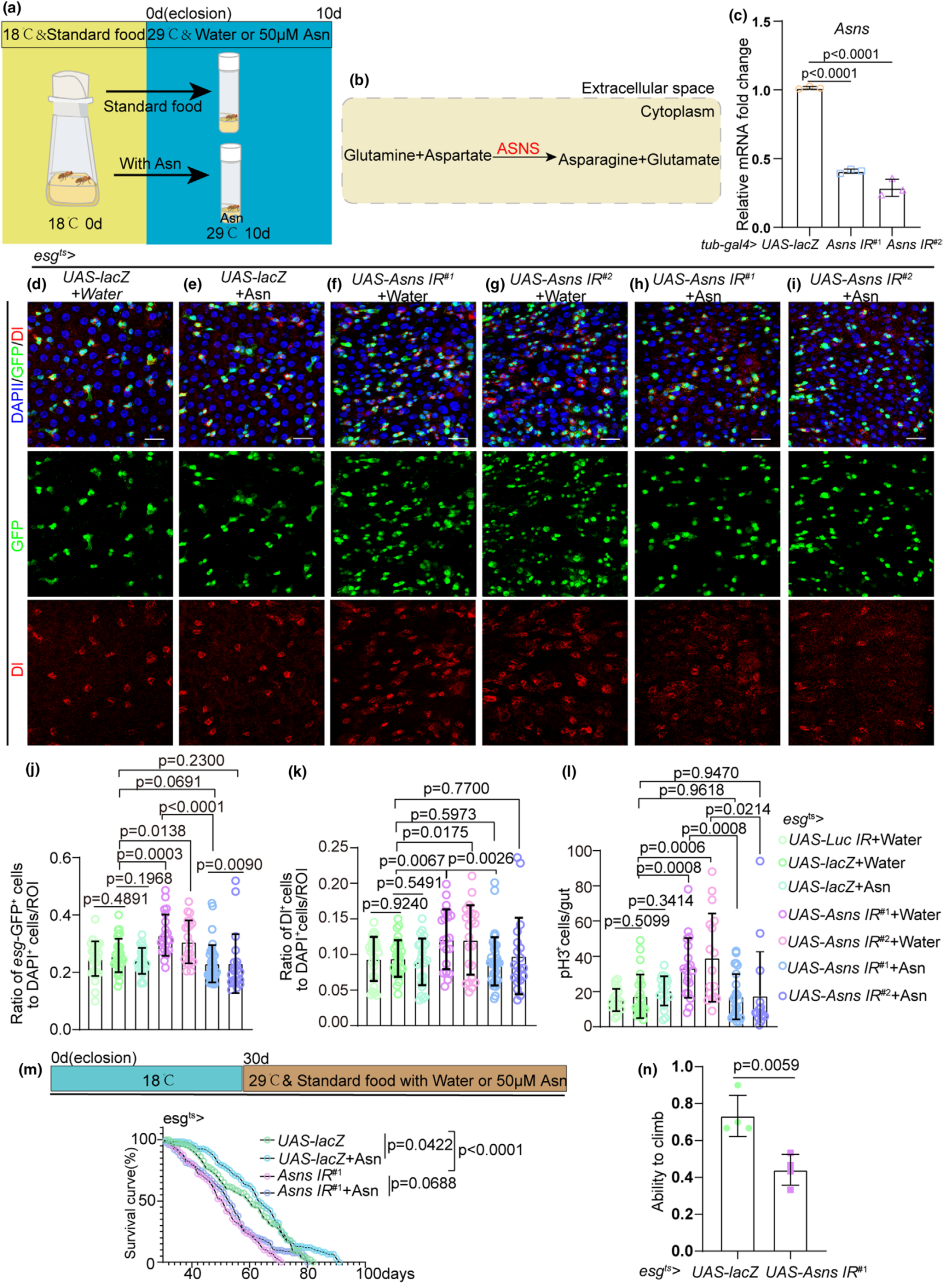
The reduced expression of ASNS in the ISCs leads to the accumulation of ISCs and Asn Extends the Lifespan in *Drosophila*. (a) Time node of feeding Asn and operation of the temperature‐sensitive Gal4 (*esg* ^*ts*^ *‐Gal4*)‐mediated system in *Drosophila*. (b) Diagram of the synthesis pattern of asparagine synthetase. (c) *ASNS* transcript levels were reduced in *Asns RNAi* (BDSC, 35739) and *Asns RNAi* (v330622) flies, relative to their levels in control (*UAS‐lacZ*) flies. Error bars show the SDs of three independent experiments. (d–i) Immunofluorescence images of the midgut section from the R4 region in *Drosophila* carrying *esg* ^*ts*^ *‐GAL4*‐driven *UAS‐lacZ* (d, control), *UAS‐lacZ* with Asn administration (e, control) *Asns RNAi* (f, g), or *Asns RNAi* in response to Asn administration (h, i). *esg*‐GFP (green) indicates ISCs and their differentiating cells. Dl staining (red) was used to visualize ISCs. Scale bars represent 25 μm. (j) Quantification of the percentage of *esg*‐GFP^+^ cells in experiments (d–i). *n*: Number of ROI counted. (*n* = 27, 29, 26, 22, 22, 39, 20, from left to right). (k) Quantification of the percentage of Dl^+^ cells in experiments (d–i). *n*: Number of ROI counted. (*n* = 27, 29, 26, 22, 22, 39, 20, from left to right). (l) Quantification of the number of pH 3^+^ cells in experiments (d–i). *n*: Number of guts counted. (*n* = 19, 24, 20, 19, 17, 25, 15, from left to right). (m) Survival percentage of female *esg* ^*ts*^ *> UAS‐lacZ* flies and *esg* ^*ts*^ *> Asns RNAi* flies without or with Asn supplementation starting from eclosion (0‐day‐old). Each group had 100 flies; all flies were at 18°C before 30 days of age and 29°C after 30 days. Three independent experiments were conducted. A log‐rank test was used for lifespan analysis. (n) The ratio of good ability to climb in *Drosophila. n*: Number of tubes counted. (*n* = 4, 4, from left to right, 10 flies per tube). Error bars represent SDs. Student's *t*‐tests and specific *p* values are indicated on the graphs.

Considering the impact of Asn on aging ISCs, we have developed a keen interest in exploring the regulatory role of the ASNS gene in ISC function. We employed *esg* ^*ts*^ *‐GAL4*‐mediated RNA interference (RNAi) to knock down ASNS expression in ISCs and assess whether the ISC accumulation occurred (Figure 2a).

Asn is synthesized in vivo through the catalytic action of ASNS, which facilitates the conversion of aspartate and glutamine into Asn via an ATP‐dependent mechanism (Lomelino et al., 2017; Staklinski et al., 2023; Yuan et al., 2024) (Figure 2b). The efficiency of *Asns RNAi* was confirmed by a significant reduction in *Asns* mRNA levels (Figure 2c). In addition, we measured the Asn levels in the intestines between control flies and *esg* ^*ts*^ *> Asns IR* flies using the Asn assay kit, obviously, *esg* ^*ts*^ *> Asns IR* flies have lower Asn level (Figure S1E). We observed that ASNS knockdown in ISCs of young *Drosophila* led to ISC accumulation, a phenotype consistent across both RNAi stocks (Figure S1F, 2D,F,G,J–L), as previously observed in aged wild‐type flies (Figure 1f). Moreover, while Asn has no significant effect on wild‐type flies (Figure 2e), its supplementation completely rescued the ISC accumulation phenotype induced by ASNS knockdown in young flies (Figure 2f–l). These findings suggest that decreased ASNS expression in ISCs may contribute to the age‐related decline in ISC functionality. Given that the reduced ASNS expression in ISCs of young flies results in ISC accumulation and that Asn alleviates this ISC accumulation phenotypes, we further investigated whether the reduced ASNS expression in the ISCs influences the lifespan of *Drosophila* and whether Asn extends it. Our data indicate that ASNS knockdown in the ISCs significantly shortens the lifespan of *Drosophila* (Figure 2m, S1G). Asn supplementation, initiated in middle‐aged flies (26 days), extends the lifespan of wild‐type *Drosophila*. In addition, Asn supplementation also shows a trend toward restoring the shortened lifespan caused by *ASNS‐RNAi* (Figure 2m). Moreover, *Drosophila* with ASNS knockdown in the ISCs exhibits lower climbing ability (Figure 2n).

### 2.3 Asn prevents age‐related decline of intestinal function in *Drosophila*

Previous studies have shown that age‐related dysfunction of ISCs leads to a significant deterioration of intestinal function in *Drosophila*, including disruption of gastrointestinal acid–base homeostasis, reduced food intake and excretion, and compromised intestinal barrier integrity (Cognigni et al., 2011; Deshpande et al., 2014; Hongjie Li & Jasper, 2016). Considering that Asn can prevent age‐related ISC deterioration, we further evaluated its potential to protect intestinal function in aged *Drosophila*. In the *Drosophila* midgut, acid secretion by the copper cells region (CCR) (Dubreuil, 2004) can be visualized using bromophenol blue as the pH indicator (Hongjie Li et al., 2016). Notably, both the size and function of the CCR decrease with age, disrupting intestinal acid–base homeostasis (Hongjie Li et al., 2016). Furthermore, intestinal barrier integrity is crucial for maintaining epithelial cell homeostasis, pathogen resistance, and immune tolerance to commensal microbiota (Rera et al., 2012). To investigate the impact of Asn on intestinal barrier function, we conducted the “Smurf” assay (Rera et al., 2011, 2012), wherein flies are fed a diet containing a nonabsorbable blue dye; leakage of the dye from the intestine into other tissues indicates compromised intestinal barrier integrity (Rera et al., 2011).

**FIGURE 3:**
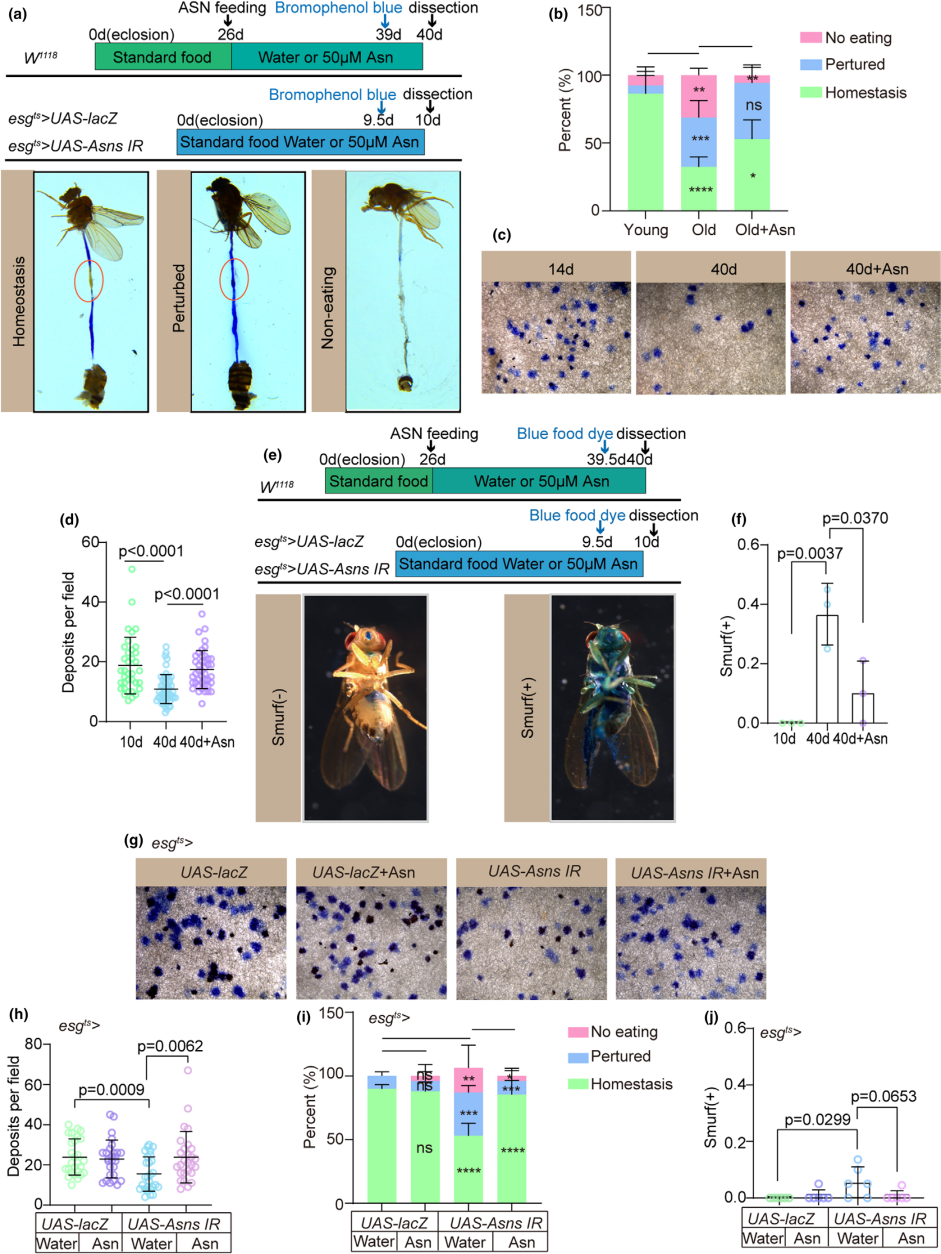
Asn prevents age‐related decline of intestinal function in *Drosophila*. (a) Representative images of the intestinal acid–base homeostasis and the noneating intestine of 10‐ and 40‐day‐old flies without Asn supplementation and 40‐day‐old flies with 0.05 mM Asn supplementation. A red ellipse indicates the CCR. “Homeostasis” refers to CCR as yellow and “Perturbed” refers to CCR as blue. “Non‐eating” means the flies did not eat, and the guts are not stained with bromophenol blue. (b) The percentage of intestinal acid–base homeostasis in experiment (a). *n* ≥ 50 flies per group. Three independent experiments were conducted. The statistical chart is based on a two‐way analysis of variance (ANOVA). The specific *p*‐values are as follows: Homeostasis (Young vs. Old: *p* < 0.0001; Old vs. Old+Asn: *p* = 0.0124). Perturbed (Young vs. Old: *p* = 0.0002; Old vs. Old+Asn: *p* = 0.7486). No eating (Young vs. Old: *p* = 0.0037; Old vs. Old+Asn: *p* = 0.0017). (c, d) Representative images (c) and quantification of excretion deposits of 10‐ and 40‐day‐old flies without Asn supplementation and 40‐day‐old flies with 0.05 mM Asn supplementation (d). Excretions are quantified in 30 fields for each group of 10 *Drosophila*. Tests were repeated as three independent experiments. *n*: Number of fields counted. (*n* = 36, 53, 43, from left to right) (e, f) Representative images (e) and the percentage of the smurf (+) flies in 40‐ and 40‐day‐old *flies* without Asn supplementation and 40‐day‐old flies with 0.05 mM Asn supplementation (f). Smurf (+) refers to a fly that leaks the blue dye from the gut into other tissues. *n* ≥ 30 flies per group. (g, h) Representative images (g) and Quantification of excretion numbers of *Drosophila* carrying *esg* ^*ts*^ *‐GAL4*‐driven *UAS‐lacZ* (control), *UAS‐lacZ* with Asn administration, *Asns RNAi*, or *Asns RNAi* with Asn administration (h). Excretions are *quantified* in 30 fields for each group of 10 *Drosophila*. Tests were repeated as three independent experiments. *n*: Number of fields counted. (*n* = 27, 27, 27, 27, from left to right) (i) Quantification of acid–base balanced intestines in *Drosophila* carrying *esg* ^*ts*^ *‐GAL4*‐driven *UAS‐lacZ* (control), *UAS‐lacZ* with Asn administration, *Asns RNAi*, or *Asns RNAi* with Asn administration. *n* ≥ 50 flies per group. Three independent experiments were conducted. The statistical chart is based on a two‐way analysis of variance (ANOVA). The specific *p*‐values are as follows: Homeostasis (*esg* ^*ts*^ *> UAS‐lacZ* vs. *esg* ^*ts*^ *> UAS‐lacZ* + Asn: *p* = 0.9833; *esg* ^*ts*^ *> UAS‐lacZ* vs. *esg* ^*ts*^ *> UAS‐Asns IR*: *p* < 0.0001; *esg* ^*ts*^ *> UAS‐Asns IR* vs. *esg* ^*ts*^ *> UAS‐Asns IR* + Asn: *p* < 0.0001). Perturbed (*esg* ^*ts*^ *> UAS‐lacZ* vs. *esg* ^*ts*^ *> UAS‐lacZ* + Asn: *p* = 0.9829; *esg* ^*ts*^ *> UAS‐lacZ* vs. *esg* ^*ts*^ *> UAS‐Asns IR*: *p* = 0.0004; *esg* ^*ts*^ *> UAS‐Asns IR* vs. *esg* ^*ts*^ > *UAS‐Asns IR* + Asn: *p* = 0.0005). No eating (*esg* ^*ts*^ *> UAS‐lacZ* vs. *esg* ^*ts*^ *> UAS‐lacZ* + Asn: *p* = 0.8841; *esg* ^*ts*^ *> UAS‐lacZ* vs. *esg* ^*ts*^ *> UAS‐Asns IR*: *p* = 0.0049; *esg* ^*ts*^ *> UAS‐Asns IR* vs. *esg* ^*ts*^ *> UAS‐Asns IR* + Asn: *p* = 0.0372) (j) quantification of the smurf (+) *Drosophila* carrying *esg* ^*ts*^ *‐GAL4*‐driven *UAS‐lacZ* (control), *UAS‐lacZ* with Asn administration, *Asns RNAi*, or *Asns RNAi* with Asn administration. Smurf (+) refers to a fly that leaks the blue dye from the gut into other tissues. *n* ≥ 50 flies per group. Three independent experiments were conducted. Error bars represent SDs. Student's *t*‐tests and specific *p* values are indicated on the graphs.

Our findings demonstrate that Asn supplementation effectively protects acid–base homeostasis in aging *Drosophila* (Figure 3a,b). Furthermore, administration of Asn from midlife significantly ameliorated the age‐related decline in food intake (Figure 3b), excretion (Figure 3c,d), and enhanced intestinal barrier function in aging flies (Figure 3e,f). Furthermore, the decrease of ASNS expression in young flies caused a decline of intestinal function like that seen in wild‐type old flies (Figure 3g–j). Asn administration entirely rescued the decline in intestinal function caused by ASNS depletion in ISCs (Figure 3g–j). In conclusion, these data support the conclusion that Asn prevents age‐induced gastrointestinal functional decline mediated by ISC aging in *Drosophila*.

### 2.4 Reduction of ASNS expression in ISCs results in the downregulation of lysosome‐ and autophagy‐related genes

**FIGURE 4:**
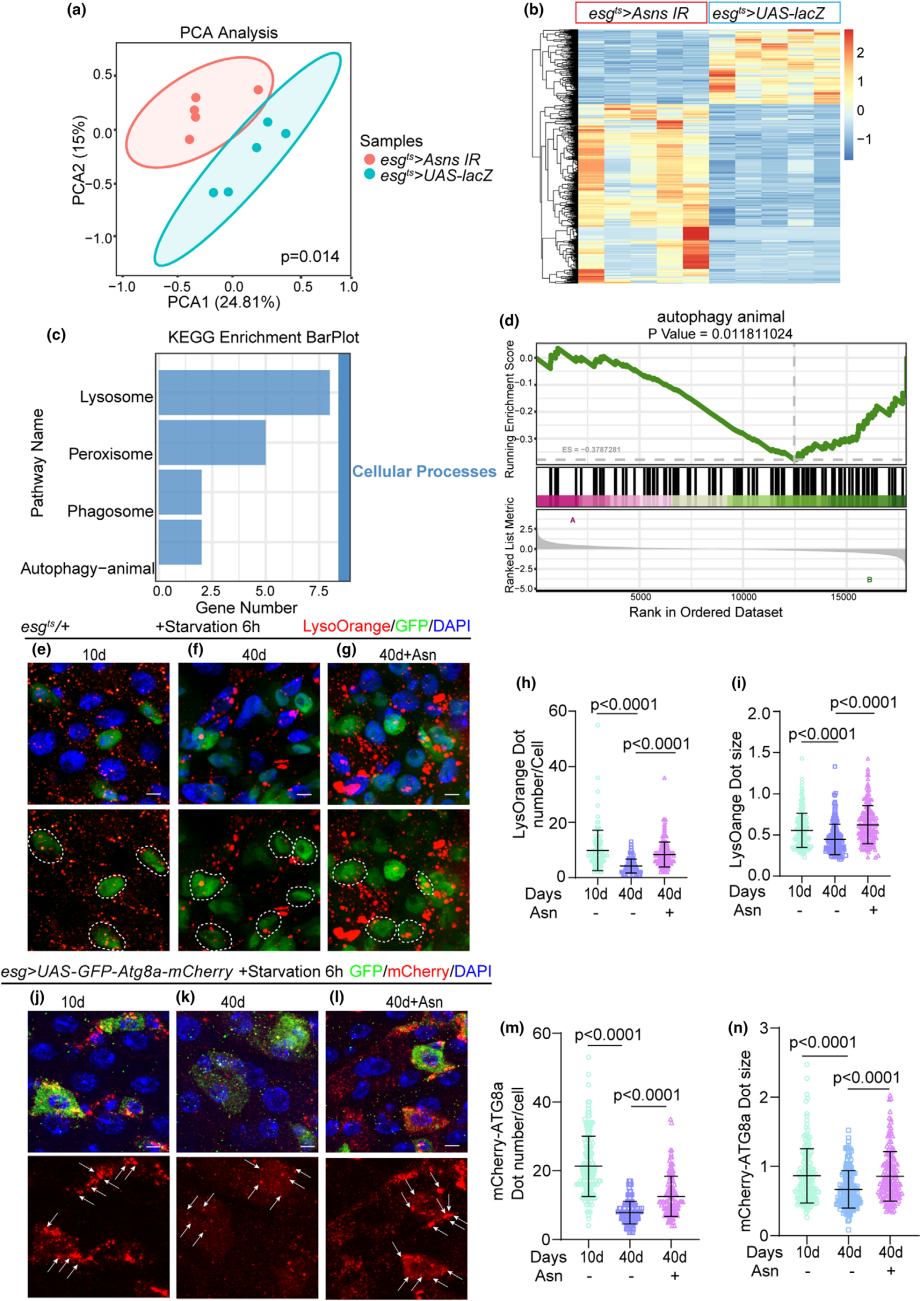
Asn regulates autophagic activation in the gut of *Drosophila*. (a) Principal component analysis (PCA) of the gut in the wild‐type group (10 days) and ASNS‐knockdown of ISCs group (10 days) of *Drosophila*. (b) The differential gene expression of *Drosophila* with ASNS‐knockdown of ISCs to wild‐type group in the heatmap. (c) The cellular process pathways of the KEGG hierarchical bar graph of the enrichment pathways influenced by ASNS knockdown in ISCs. (d) The Gene Set Enrichment Analysis (GSEA) graph on the autophagy animal, represents downregulated autophagy activity. Source data are available online for this figure. (e–g) Immunofluorescence images of *esg*‐GFP and LysoOrange staining with the midgut section from the R4 region in 10‐day *esg* ^ts^/+ flies (e), 40‐day *esg* ^ts^/+ flies (f), 40‐day *esg* ^ts^/+ flies with Asn administration (g). *esg*‐GFP (green; outlined by dotted lines), LysoOrange (red). *esg*‐GFP^+^ cells are outlined by white dotted lines in the images of the LysoOrange staining channel. (h) Quantification of the dot number of LysoOrange in *esg*‐positive cells from experiments (e–g). *n*: Number of *esg*‐positive cells counted. (*n* = 103, 141, 217, from left to right) (i) Quantification of the dot size of LysoOrange in *esg*‐positive cells from experiments (e–g). *n*: Number of dots measured. (*n* = 190, 186, 174, from left to right) (j–l) Expression of *esg‐GAL4*‐driven *UAS‐GFP‐mCherry‐Atg8a* in 10‐day *Drosophila* (j), 40‐day *Drosophila* (k), 40‐day *Drosophila* with Asn administration started in middle age (26 days) (l). GFP (green) and mCherry (red). The white arrows indicate the autophagosomes. (m) Quantification of the dot number of mCherry in *esg*‐positive cells from experiments (j–l). *n*: Number of *esg*‐positive cells counted. (*n* = 160, 152,134, from left to right) (n) Quantification of the dot size of mCherry in *esg*‐positive cells from experiments (j–l). *n*: Number of dots measured. (*n* = 166, 172, 163, from left to right).

To explore the underlying mechanism of ASNS in maintaining the homeostasis of *Drosophila* ISCs, we conducted RNA‐sequencing (RNA‐seq) analysis of the midguts of *Drosophila esg* ^+^ cells both with and without ASNS knockdown. Principal component analysis (PCA, Figure 4a) disclosed a pronounced divergence between the control group and the ASNS‐knockdown group, signifying the group of ASNS‐knockdown substantial impact on the transcriptome of the *Drosophila* midgut. According to the Kyoto Encyclopedia of Genes and Genomes (KEGG) enrichment ES line graph of the GSEA analysis, there was a decrease in the metabolism of aspartate and glutamine, reflecting the indeed downregulated expression of ASNS in the midguts of the experimental group of flies (Figure S2A). From the heatmap results (Figure 4b), it is evident that there were significant differences in gene expression between the ASNS‐deprived group of flies and the control group of flies, including 786 upregulated genes and 340 downregulated genes, also seen in a Venn diagram (Figure S2B). Through KEGG pathway enrichment analysis, we observed in the KEGG hierarchical bar graph that lysosome‐related genes show the most significant differential expression in the cellular process pathway of differentially expressed genes, with autophagy‐related genes also clustering in this bar (Figure 4c). The autophagic lysosomal pathway plays a crucial role in cellular, tissue, and organismal homeostasis (Levine & Kroemer, 2019), with lysosomes as key nodes between autophagy and endocytosis, playing indispensable roles in various types of autophagic processes (Mahapatra et al., 2021). Therefore, we have a keen interest in the autophagic lysosomal pathway. Furthermore, the KEGG enrichment ES line graph of the GSEA analysis about the autophagy‐animal pathway showed the autophagy downregulated in the ASNS‐knockdown group (Figure 4d). Additionally, from the volcano plot, we found that some genes associated with lysosome genes were downregulated in the intestines of ASNS‐knockdown *Drosophila* (Figure S2D). Furthermore, the KEGG enrichment ES line graph of the GSEA analysis indicated the longevity regulation pathway shows downregulation in the ASNS‐knockdown group (Figure S2C), consistent with our previous experimental results (Figure 2m, S1G). These results further support the important role of ASNS in maintaining homeostasis of *Drosophila* ISCs and provide clues for further research on the regulatory mechanisms of ASNS in autophagy‐ and lysosome‐related pathways.

### 2.5 Asn regulates autophagic activation in the *Drosophila* intestine

Over the years, autophagy activation has been intimately associated with anti‐aging (Kaushik et al., 2021; Martinez‐Lopez et al., 2015; Revuelta & Matheu, 2017), and both in vitro and in vivo models have revealed the pivotal role of autophagy in maintaining intestinal homeostasis (Foerster et al., 2021; Larabi et al., 2019). Our previous RNA sequencing analysis revealed that ASNS depletion in *esg* ^+^ cells is associated with the difference in lysosomal and autophagy‐related genes compared to the control group. Consequently, we confirmed the potential regulatory role of Asn in autophagy. To visualize autophagic activity in ISCs, we employed LysoOrange, an acidophilic dye used to detect autophagy‐related lysosomal activity, and the ATG8a‐GFP‐mCherry reporter gene, which measures dynamic autophagic flux (Mauvezin et al., 2014; Valko et al., 2021). We examined ISCs of aged intestines with and without Asn administration. Analysis using LysoOrange revealed significantly higher autophagic activity in young ISCs (10 days; Figure 4e,h,i) compared to aged ISCs (40 days; Figure 4f,h,i). Asn markedly increased autophagic activity in aged ISCs (Figure 4g–i). Similarly, *Drosophila* carrying UAS‐GFP‐mCherry‐Atg8a driven by *esg‐gal4* displayed more numerous and larger mCherry foci in young ISCs compared to aged ISCs (Figure 4j,k,m,n). Furthermore, Asn significantly enhanced autophagic activity in ISCs of aged flies, as evidenced by the formation of more numerous and larger mCherry dots (Figure 4j–n).

Furthermore, the reduction of ASNS in ISCs of young flies via *Asns RNAi* mediated by *esg* ^*ts*^ *‐gal4* resulted in a significant decrease in LysoOrange signals (Figure S2E–H). Furthermore, the reduction of ASNS in ISCs significantly decreased autophagic activity in ISCs of aged flies, as evidenced by the formation of fewer and smaller mCherry dots (Figure S2I–L). We speculated that ASNS depletion in *esg* ^+^ cells may induce premature aging of the entire midgut.

### 2.6 Asn rescues the accumulated phenotype of aging ISCs by activating autophagy

**FIGURE 5:**
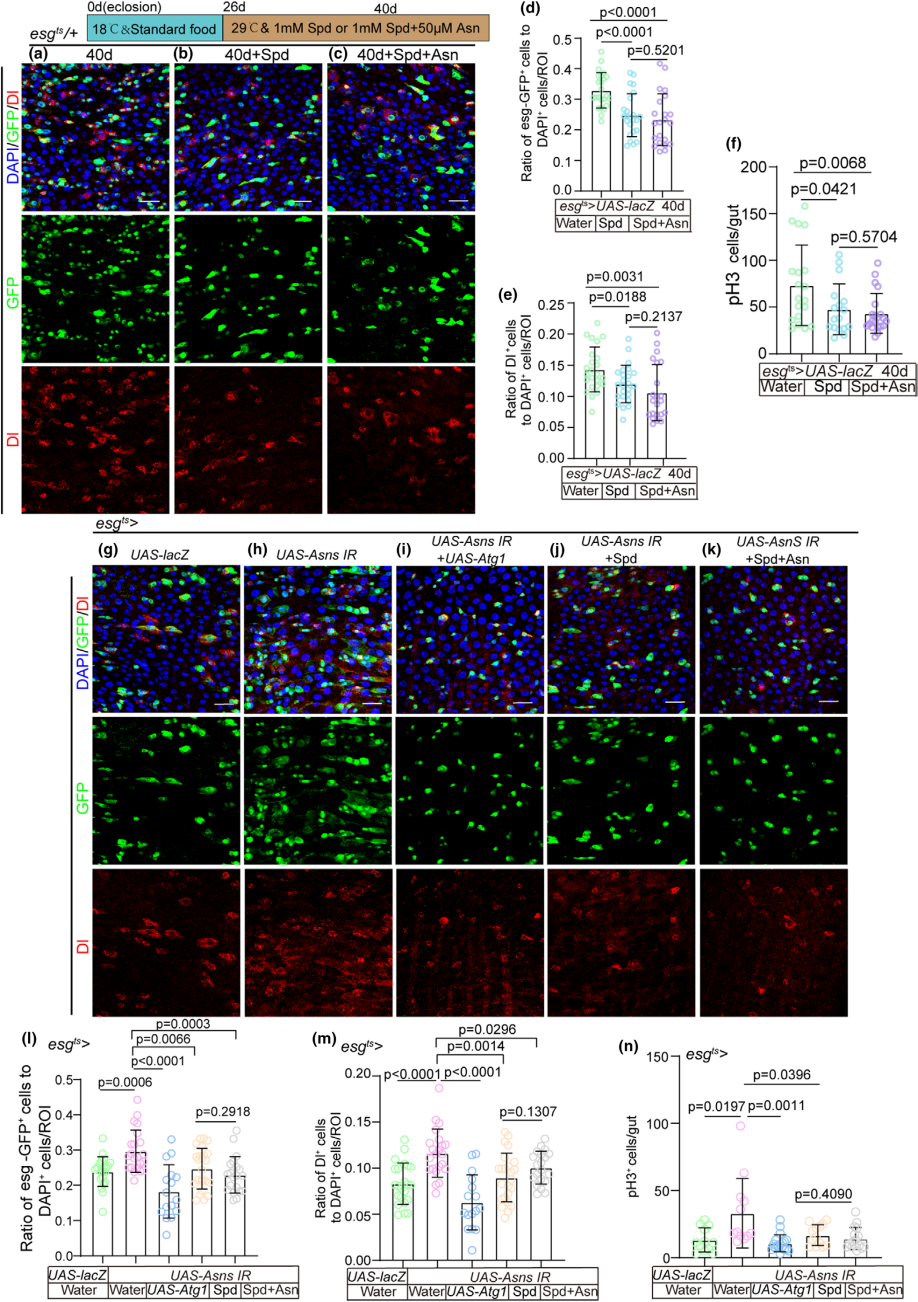
Asn does rescue the accumulated phenotype of aging ISCs by activating autophagy. (a–c) Immunofluorescence images of midgut section from the R4 region in 40‐day flies (a), 40‐day flies with spermidine (Spd) administration started at 26th day after fly eclosion (b), 40‐day flies with Spd and Asn administration started at 26th day after fly eclosion (c). *esg*‐GFP (green) indicates ISCs and their differentiating cells. Dl (red) staining was used to visualize ISCs. (d) Quantification of the percentage of *esg*‐GFP^+^ cells in experiments (a–c). *n*: Number of ROI counted. (*n* = 24, 24, 22, from left to right). (e) Quantification of the percentage of Dl^+^ cells in experiments (a–c). *n*: Number of ROI counted. (*n* = 24, 24, 22, from left to right). (f) Quantification of the number of pH 3^+^ cells in experiments (a–c). *n*: Number of guts counted. (*n* = 19, 17, 21, from left to right). (g–k) Representative images of the midgut section from the R4 region of *Drosophila* carrying *esg* ^*ts*^ *‐GAL4*‐driven overexpression of lacZ cDNA (g, control), *Asns RNAi* (h), *Asns RNAi* and Atg1 cDNA (i), *Asns RNAi* with Spd administration (j), or *Asns RNAi* with Spd and Asn administration (k). GFP (green) and Dl staining (red) were used to visualize ISCs. (l) Quantification of the percentage of *esg*‐GFP^+^ cells in experiments (g–k). *n*: Number of ROI counted. (*n* = 22, 22, 16, 24, 21, from left to right). (m) Quantification of the percentage of Dl^+^ cells in experiments (g–k). *n*: Number of ROI counted. (*n* = 23, 22, 16, 24, 20, from left to right). (n) Quantification of the number of pH 3^+^ cells in experiments (g–k). *n*: Number of guts counted. (*n* = 12, 12, 19, 13, 16 from left to right). Three independent experiments were conducted. Error bars represent SDs. Student's *t*‐tests and specific *p* values are indicated on the graphs.

Spermidine (Spd), a natural polyamine essential for cellular homeostasis and growth, has been demonstrated to promote autophagy (Hofer et al., 2021), extend the lifespan of diverse organisms, and mitigate various age‐associated disorders in murine models (Hofer et al., 2022). Previous studies have established that either feeding aging *Drosophila* Spd or overexpression of ATG1 (a protein known for its regulatory role in initiating autophagy) is sufficient to induce autophagy (Neufeld, 2007; Tang et al., 2011; Wang et al., 2024). In our study, we observed that feeding aging *Drosophila* Spd significantly suppressed the phenotype of ISC accumulation (an increase of *esg*‐GFP^+^ cells, Dl^+^ cells, and pH 3^+^ cells in midguts) (Figure 5a,b,d–f). Furthermore, Asn could not further reduce this phenotype in aging flies with Spd‐activated autophagy (Figure 5c–f). The ISC accumulation phenotype induced by ASNS depletion in ISCs was largely rescued by either Spd administration or ATG1 overexpression (Figure 5g–j,l–n). However, Asn did not further decrease this phenotype (Figure 5k–n). Moreover, ATG8a depletion in *esg* ^+^ cells induced an ISC accumulation phenotype that Asn was unable to rescue (Figure S3A–F). These findings suggest that autophagy acts downstream of Asn in the molecular pathway that prevents the functional decline of ISCs in response to aging. The data supports a model wherein Asn exerts its beneficial effects on aging ISCs primarily through the activation of autophagy, underscoring the critical role of this cellular process in maintaining ISC homeostasis and function during aging.

### 2.7 Asn restrains ISC overproliferation in old *Drosophila* by a TOR‐independent, MAPK‐mediated autophagy signaling pathway

We have found that Asn rescues the accumulated phenotype of aging ISCs by activating autophagy. We are curious about how Asn activates autophagy in aging *Drosophila*. Given that previous studies have implicated the Mitogen‐activated protein kinases (MAPK) signaling pathway as a major trigger for ISC overproliferation when the autophagy network is perturbed (Zhang et al., 2019). Therefore, we focused on this pathway, one of the TOR‐independent upstream signaling pathways of autophagy, MAPK (Al‐Bari & Xu, 2020; Cao et al., 2021). In this study, we assessed MAPK activity in aging ISCs in response to Asn administration or not by monitoring the levels of phosphorylated ERK (pERK). We found that Asn administration significantly reduced MAPK signaling activation in ISCs of old flies (Figure S3G–I). Conversely, the depletion of ASNS in ISCs leads to a significant upregulation of MAPK activity (Figure S3J–L). In summary, Asn mitigates ISC overproliferation in aging *Drosophila* by activating autophagy via inhibiting MAPK signaling.

### 2.8 Asn can enhance the functionality of aged ISCs in murine intestinal organoids

**FIGURE 6:**
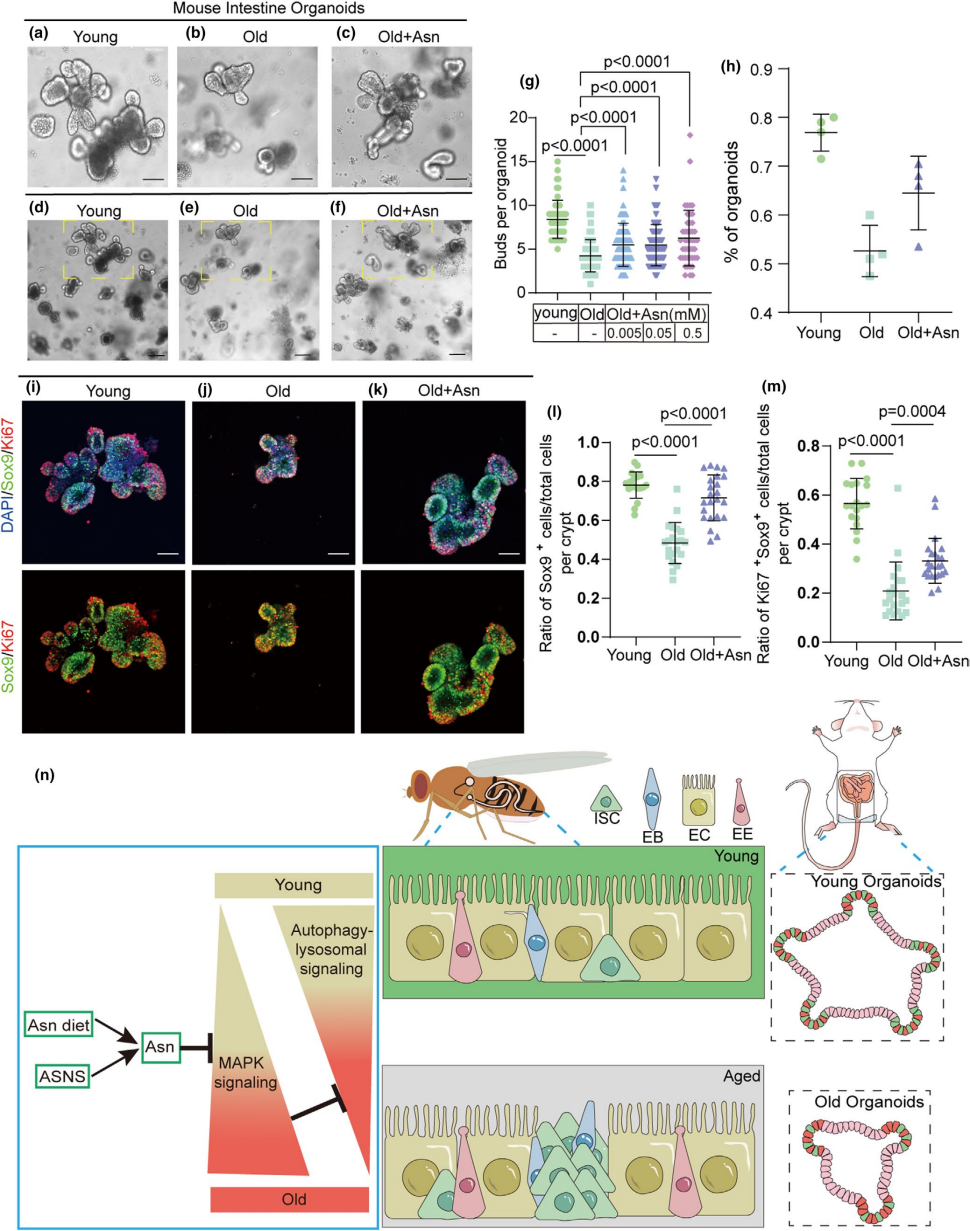
Asn can enhance the function of aged ISCs in mouse organoids. (a–f) Representative images of young mouse organoids and old mouse organoids treated or not with Asn. (a–c scale bar: 150 μm; d–f scale bar: 100 μm) (g) Quantification of the buds of small intestinal organoids. n corresponds to one organoid. (*n* = 55, 111, 80, 61, 46, from left to right). (h) Quantification of the formation rates of small intestinal organoids. n corresponds to one well with cultured organoids. Each group had 4 wells. (i–k) Representative immunofluorescence images of young mouse organoid and old mouse organoid treated or not with Asn. Stained with DAPI (blue), SOX9 (green; ISC marker), and Ki67 (red; proliferating cell marker). Scale bars represent 75 μm. (l) The ratio of SOX9^+^ cells to total cells per crypt in experiments i–k. *n*: Number of organoids counted. (*n* = 19, 21, 23, from left to right). (m) The ratio of Ki67^+^SOX9^+^ cells to total cells per crypt in experiments i–k. *n*: Number of organoids counted. (*n* = 19, 21, 23, from left to right). Three independent experiments were conducted. Data information: Error bars represent SDs. Student's *t*‐tests and specific *p* values are indicated on the graphs. (n) Graphical summary of Asn rejuvenates ISC aging.

Complementing *Drosophila* research, the mouse intestinal organoid model, whose formative capacity heavily relies on ISC activity (Zhao et al., 2020), has been used for investigating mammalian intestinal aging and ISC functionality (Clevers, 2016; Jasper, 2020; Lancaster & Knoblich, 2014; Zhao et al., 2020). In mammals, aging intestinal crypts exhibit reduced numbers, delayed proliferation, and increased cell apoptosis (Martin et al., 1998). Correspondingly, the aging organoid formation and budding frequency of ex vivo intestinal organoids are also reduced (Nalapareddy et al., 2017). To further confirm the anti‐aging effect of Asn, we quantified the organoid formation frequency in both young and aged murine intestinal crypts. After 7 days of culture, we observed a significant decrease in both budding and formation rates in the aged organoids (Figure 6a,b,d,e). However, treatment of organoids derived from aged murine intestines with 0.05 mM Asn resulted in a marked increase in both the number of organoids and the count of leaflet/bud per organoid (Figure 6b–h). These findings suggest that Asn supplementation improves the functionality of aged organoids.

To provide further evidence of the anti‐aging effects of Asn in intestinal organoids, we employed SOX9 and Ki67 as markers to label ISCs and proliferating cells. Results indicated a significant increase in the number of SOX9^+^ cells and Ki67^+^SOX9^+^ cells in each crypt of Asn‐treated aged intestinal organoids compared to untreated aged organoids (Figure 6i–m). Collectively, these results demonstrate that Asn treatment may alleviate age‐induced ISC functional decline in murine intestinal organoids.

## 3 DISCUSSION

Over the past few decades, research in the field of aging has intensely focused on determining efficient treatment strategies to delay aging and reduce aging‐related diseases. This study has identified that Asn, a nonessential amino acid, exerts excellent effects in maintaining intestinal homeostasis and extending the lifespan of aged *Drosophila*. Previous studies on Asn and the gene *ASNS* predominantly centered on their roles in tumor biology (Halbrook et al., 2022; Jiang et al., 2021; Knott et al., 2018; Krall et al., 2021; H. Li et al., 2019). However, other functions of Asn, especially in the context of aging, have been largely overlooked. Herein, we found that Asn prevents the age‐associated functional decline of ISCs in aged *Drosophila* via activating the autophagy‐lysosomal pathway by inhibiting the MAPK signaling pathway (Figure 6n).

Aging is characterized by the progressive decline in organism functions, driven by stem cell aging, which increases susceptibility to various diseases. In *Drosophila*, the ISCs exhibit hyperproliferation during aging, disrupting intestinal homeostasis and impairing intestinal function (Jasper, 2020). Our study represents a pioneering investigation into the role of Asn in stem cell‐mediated tissue degeneration and organismal aging. We demonstrate that oral administration of Asn significantly attenuates ISC hyperproliferation and gut functional decline in both aged wild‐type *Drosophila* and young flies with ASNS depletion in ISCs. While, in *ASNS‐RNAi*‐treated flies, the beneficial effects of Asn on intestinal health and ISC function were accompanied by a trend toward restoring lifespan, though this trend did not reach statistical significance (Figure 2m). The potential reason for this result may be that the lifespan of *Drosophila* is influenced by a variety of factors (Bylino et al., 2024). Additionally, the relationship between gut health and lifespan is complex (Norman & Klaus, 2020), involving multiple biological processes, such as metabolism, apoptosis, and immune responses. Although Asn can improve gut phenotypes, these improvements may not be sufficient to result in a significant extension of lifespan. Moreover, we further validated that Asn treatment alleviated age‐induced decline in murine intestinal organoids. However, the underlying mechanisms by which Asn improves the function of intestinal organoids in aging mice need further investigation.

Our research may provide potential evidence for Asn to promote intestinal health in aging populations, where decreased intestinal permeability (DeJong et al., 2020) and impaired nutrient absorption (Woudstra & Thomson, 2002) are common. We found that Asn may strengthen the intestinal barrier (Figure 3e, f), potentially reducing the risk of conditions like leaky gut syndrome (Kavanagh et al., 2019) often observed in the elderly. Additionally, as a nonessential amino acid, Asn is widely used to produce other essential nutrients such as glucose, proteins, lipids, and nucleotides (Yuan et al., 2024), improving overall nutritional status and health. Therefore, Asn may serve as a therapeutic agent in the future, potentially acting as a dietary supplement to support gut health in older adults and mitigate age‐related declines in gut function, ultimately enhancing quality of life.

Autophagy is a key intracellular degradation system by which cytoplasmic materials are delivered to and degraded in the lysosome (Mizushima & Komatsu, 2011). It serves a critical housekeeping role by eliminating misfolded or aggregated proteins, damaged organelles, and intracellular pathogens (Glick et al., 2010). Suppression of autophagy causes age‐dependent dysfunction in various organs (Mizushima & Komatsu, 2011). Previous research in vitro hepatocyte systems found that Asn might inhibit autophagy (Høyvik et al., 1991). However, contrary to this conclusion, our RNA‐seq data in live‐fly models revealed that knocking down ASNS in ISCs downregulates the autophagy pathway. We also observed reduced autophagic activity in the intestines of flies with ASNS knocked down in ISCs. Furthermore, we confirmed that feeding Asn to aging flies activates autophagy in ISCs. The reason for this difference may be that Asn regulates autophagy differently in various cell types and under different physiological conditions. We believe that once Asn is absorbed into the body, it is involved in a series of complex metabolic processes, but further research is needed to determine which specific metabolic steps promote the autophagy pathway.

The Mitogen‐activated protein kinases/extracellular signal‐regulated kinase (MAPK/ERK) pathway is reported to be associated with cell proliferation (Fang & Richardson, 2005), differentiation, migration, senescence, and apoptosis (Sun et al., 2015). MAPK, one of the mTOR‐independent pathways, acts upstream of autophagy (Al‐Bari & Xu, 2020; Cao et al., 2021) and is hyperactivated in aging (He et al., 2020). Our study found that both aged wild‐type and young fly midguts with ASNS depletion in ISCs in *Drosophila* exhibited over‐activation of MAPK signaling. The molecular mechanisms underlying the anti‐aging effects of Asn appear to be mediated through the modulation of the MAPK and autophagy signaling pathways. We posit that Asn improves the function of aging ISCs by activating autophagy through inhibiting MAPK activity. There is a need for further studies to elucidate the specific mechanism through which Asn regulates the pathway.

While we have validated Asn's phenotypic effects in aging flies using mouse organoids, future research needs to conduct in vivo validation of our findings in mammalian models. Therefore, these findings provide preliminary insights into gut health but cannot yet be directly translated into clinical applications at this stage. Future research may involve assessing the impact of Asn on gut health and aging states in mammals, bridging the gap between organoid studies and clinical applications. Additionally, it is crucial to collaborate with clinical researchers to design studies that can effectively translate our findings into potential therapeutic strategies for human gut health.

## 4 MATERIALS AND METHODS

### 4.1 *Drosophila* culture and stocks

All flies were maintained on standard food with cornmeal‐agar (including 80 g sucrose, 50 g cornmeal, 20 g glucose, 18.75 g yeast, 5 g agar, and 30 mL propionic acid dissolved in 1 L water) and kept at standard conditions (25°C, 60% relative humidity, and 12 h light/dark cycle) unless otherwise mentioned. In this study, all flies used were mated females. In addition, at room temperature, young flies were between 10 and 14 days, middle‐aged flies were about 26 days, and aging flies were approximately 40 days. Moreover, all the *Drosophila* used were backcrossed for 5–6 generations to eliminate their genetic background.

The following *Drosophila* lines were used: *esg‐GFP/CyO* line and *esg‐GAL4* line (from Allan Spradling), *esg* ^*ts*^ *‐Gal4* line (from Benjamin Ohlstein), *UAS‐lacZ* line and *tub‐Gal4* line (from Allan Spradling), *w* ^*1118*^ line (BDSC# 3605), *Asns RNAi* line (BDSC# 35739; VDRC# v330622), *UAS‐GFP‐mCherry‐Atg8a* line (BDSC# 37749), *UAS‐Atg1* line (BDSC# 51655), *Atg8a RNAi* (BDSC# 34340), *UAS‐LUC RNAi* (BDSC# 31603), *UAS‐Asns* (self‐constructed). The *Drosophila* genotypes used in this study are listed in Table S1.

In this study, the UAS/Gal4 system was used. Noticeably, knockdown or overexpression of genes was repressed at 18°C and activated at 29°C through the temperature‐sensitive Gal4 (*esg* ^*ts*^ *‐Gal4*)‐mediated system.

### 4.2 Asparagine treatments

Asparagine (Macklin, Shanghai, China, 70–47‐3) was first dissolved in water and then added to standard food and mixed completely to obtain different concentrations (0.005, 0.05, 0.5, 5 mM). Flies were collected randomly and distributed equally into mixed food vials containing Asn or water (as control).

### 4.3 Asparagine content determination

The concentration of ASN in the samples was measured using a commercially available ASN assay kit (Catalog No. G0437W, Suzhou Grace Biotechnology Co., Ltd.) according to the manufacturer's instructions. First, the intestines of *Drosophila* were dissected, weighed, and thoroughly homogenized. Then, samples and reagents were sequentially added to a 96‐well plate according to the kit instructions. The mixture was thoroughly mixed and incubated at room temperature (25°C) for 10 min. Absorbance was measured at 340 nm using a microplate reader, and the ASN concentration was calculated using the formula provided in the kit instructions.

### 4.4 Immunofluorescence and microscopy for *Drosophila* midguts

The guts of flies were dissected in cold 1 × phosphate‐buffered saline (PBS), this dissected fly guts were fixed for 30 min with 4% paraformaldehyde (PFA) at room temperature with shaking and then washed three times in 0.1% PBST (PBS containing 0.1% Triton X‐100 (Aladdin, #T109027)) for 10 min each. Next, the tissues were blocked with 0.5% BSA for 30 min and incubated with the primary antibodies diluted in 0.1% PBST overnight at 4°C. On the second day, the guts were incubated with secondary antibodies and DAPI for 2 h at room temperature with shaking followed by the same washing steps above. Finally, the guts were washed three times in 0.1% PBST for 10 min each. Samples were mounted on glass slides for microscopy after washing.

The primary antibodies and dilutions used in this study were shown in Table S2. The secondary antibodies and dilutions were as follows: Goat anti‐chicken Alexa 488 (Invitrogen, Shanghai, China, 1:2000), Goat anti‐mouse Alexa 568 (Invitrogen, 1:2000), and Goat anti‐rabbit Alexa 568 (Invitrogen, 1:2000) antibodies. The nuclei staining was performed with 1 μg/mL of 4, 6‐diamidino‐2‐phenylindole (DAPI; Sigma, Shanghai, China).

All immunofluorescence images were acquired using a Leica TCS‐SP8 confocal microscope and then subsequently analyzed with Leica Application Suite X (LAS X, Weztlar, Germany), and Adobe Illustrator.

### 4.5 Climbing assay

Female flies of different ages were transferred to empty vials separately, and a line was drawn 10 cm from the bottom, with each group containing 30 to 40 flies. The vials were tapped three times to ensure that all flies were at the bottom and remained vertical for about 15 s. Videos were recorded, and the number of flies crossing the 10‐cm line within 10 s was analyzed. This measurement was repeated three times, and the mean value was calculated.

### 4.6 Lifespan assay

To determine the effect of Asn and ASNS on the lifespan, 100 *esg* ^*ts*^/+ flies were gathered and randomly distributed into five vials, with and without Asn. Similarly, 100 *esg* ^*ts*^ *> ASN* *S* *RNAi* flies were subjected to the same grouping procedure. The mortality of the flies was documented every one or 2 days, with vials being replaced as needed. This experiment was repeated three times for robustness and reliability.

### 4.7 Nucleic acid extraction and RT‐qPCR

Each experiment involved the collection of 20 adult midguts, preserved in DEPC‐treated PBS solution at 4°C. RNA extraction was carried out from the samples using the RN07‐EASYspin Fast Pure Cell/Tissue Total RNA Isolation Kit (Aidlab, #RN0702), followed by reverse transcription using the Evo M‐MLV RT Kit (Accurate Biology, #AG11711).

Subsequent RT‐qPCR analysis was performed using the ChamQ Universal SYBR qPCR Master Mix (Vazyme, #Q311) on the CFX96 Touch™ Real‐time PCR System (Bio‐RAD, Hercules, USA). The relative expression levels of mRNA of the target genes were quantified using the *rp49* (*Drosophila*) gene as a reference control, employing the 2^−ΔΔCT^ method. Primer sequences for the genes used are listed in Table S3.

### 4.8 Bromophenol blue assay

A protocol previously outlined in reference (Hongjie Li et al., 2016) was employed to conduct the bromophenol blue assay, aimed at assessing the normalcy of the acidic state within the copper cell region (CCR) in the *Drosophila* gut. Specifically, 200 μL of 2% bromophenol blue sodium (Sigma, #B5525) was carefully dispensed onto the surface of the food medium, ensuring adequate permeation by creating multiple holes. Subsequently, 30 flies per vial underwent a 2‐h starvation period before being exposed to the prepared food for 24 h. Finally, the guts were dissected and promptly imaged. This experimental procedure was repeated three times for each test group.

Upon ingestion of food infused with bromophenol blue, flies exhibited blue staining in their guts, characterized as “Eating” flies. Conversely, a “Non‐eating” fly refers to one that abstained from consuming the food and consequently did not exhibit blue staining in its gut. The percentage of guts displaying blue staining was quantified as “Eating (%)”.

### 4.9 Fly excretion measurement

The flies underwent 2 h of food deprivation and were then fed for 24 h with bromophenol blue‐infused food. The resulting deposits, where each blue dot denoted a deposit, left by the flies on the vial walls were carefully collected onto chromatography paper. These deposits on the paper were then meticulously observed and tallied using a Leica M205 FA stereomicroscope (Leica, Weztlar, Germany) to quantify the number of deposits.

### 4.10 Smurf assay

The Smurf assay was conducted to assess the integrity of the intestinal barrier following the established protocol. A 2.5% (wt/vol) brilliant blue solution (Aladdin, # B295002) was incorporated into the standard food. Thirty flies per vial underwent a 2‐h starvation period and were then placed in the prepared medium for 24 h. Flies displaying blue dye outside their digestive tracts were identified as “Smurf (+)”. This experiment was repeated three times for each test group.

### 4.11 Midgut dissection for RNA‐seq

The midguts of adult flies were initially dissected in a cold DEPC‐PBS solution. The midguts of adult flies (R1‐R5) were dissected to separate them from the foreguts, hindguts, Malpighian tubules, and trachea. Then, the total RNA was then extracted from more than 50 female midguts (R1‐R5) for RNA‐seq analysis using TRIzol Reagen (thermofisher, 15,596,018). The total RNA quantity and purity were analyzed with Bioanalyzer 2100 and RNA 6000 Nano LabChip Kit (Agilent, CA, USA, 5067–1511), and high‐quality RNA samples with RIN number >7.0 were used to construct a sequencing library. After total RNA was extracted, mRNA was purified from total RNA (5 μg) using Dynabeads Oligo (dT) (Thermo Fisher, CA, USA) with two rounds of purification.

A cDNA library was sequenced and run with the Illumina NovaseqTM6000 sequence platform. Using the Illumina paired‐end RNA‐seq approach, the transcriptome was sequenced, generating a total of million 2 × 150 bp paired‐end reads. Reads obtained from the sequencing machines include raw reads containing adapters or low‐quality bases which will affect the following assembly and analysis. Thus, to get high‐quality clean reads, reads were further filtered by Cutadapt (https://cutadapt.readthedocs.io/en/stable/, version: cutadapt‐1.9).

The genes with the parameter of false discovery rate (FDR) below 0.05 and absolute fold change ≥2 were considered differentially expressed genes. Differentially expressed genes were then subjected to enrichment analysis of GO functions and KEGG pathways. The raw sequence data of this study have been submitted to the NCBI Sequence Read Archive (SRA) datasets with BioProject ID PRJNA1138061.

### 4.12 CytoPainter LysoOrange staining

Midguts were dissected and then incubated with CytoPainter LysoOrange Indicator Reagent (1:500, Abcam, ab176827) for 30 min, followed by Hoechst for 10 min. The midguts were washed three times for 2 min each with PBS and imaged immediately by confocal microscopy. All washes and staining steps were performed at room temperature and in a dark environment.

### 4.13 Fluorescence intensity statistics

Immunofluorescence imaging results were analyzed using z‐stacks obtained with the Leica TCS‐SP8 confocal microscope. Fluorescence intensity of the region of interest (ROI) was calculated by LAS X software. The ROI was selected using Stack Profile tools in the Quantify interface, and a smaller surrounding region was chosen as the background. Different ROIs corresponding to dpERK in ISCs, and background regions were defined. By checking the “Statistics” boxes, the integrated density was calculated as follows: Integrated Density = Integrated Density of ROI—Integrated Density of background region/Area of background region × Area of ROI.

### 4.14 Crypt isolation, organoid culture

Mouse intestines were rinsed with cold PBS to remove fecal material, and then opened laterally with scissors. The villi were gently scraped with a glass slide. The cleaned intestine was cut into small segments with tweezers, then transferred to a 50 mL centrifuge tube and washed with 15 mL of cold DPBS for 10–15 cycles until the supernatant appeared clear. The remaining tissue was then cut into 1–2 cm sections, thoroughly flushed, and incubated with 25 mL of cold DPBS containing 5 mM EDTA on a shaker at 20 rpm at 8°C for 1.5 h after discarding the supernatant. Subsequently, the tissue was mechanically shaken and filtered through a 70‐μm mesh to remove any villous fragments. The resulting crypts were harvested into a fresh 50 mL centrifuge tube and kept on ice. This process was repeated to collect 2–4 sections. Fifty microliters from each fraction were placed into a well of a 24‐well plate to estimate crypt numbers under a microscope, and the fraction with the highest concentration of crypts was selected. This fraction was centrifuged at 290 g for 5 min at 2–8°C, the supernatant was discarded, and the pellet was retained. The pellet was resuspended in 10 mL of cold DPBS with 0.1% BSA and centrifuged at 200 g for 3 min at 2–8°C to remove the supernatant, leaving the cryps. The crypts were then resuspended in 10 mL cold DMEM/F‐12, and crypt density was calculated in units of 10 μL aliquots (15 cryps/10 μL is approximately equal to 1500 cryps/mL). The isolated crypts were quantified and combined with a 1:1 mixture of medium and Matrigel (Gibco, #A1413202). The concentration was adjusted by centrifugation to 15–25 crypts per μL for cultivation in a 24‐well plate. If not otherwise, the crypts were cultured in a blend of Advanced DMEM/F12 medium (Gibco, #10565018) and IMDM medium (Gibco, #31980097), supplemented with EGF at 40 ng/mL (Peprotech, #315–09), Noggin at 200 ng/mL (Peprotech, #250–38), R‐Spondin at 500 ng/mL (Peprotech, #120–38), N‐acetyl‐L‐cysteine at 1 mM (Sigma Aldrich, #A7250), B27 at 2× (Gibco, #17504044), and N2 at 1× (Gibco, #17502001). The intestinal crypts were dispensed in 50 μL Matrigel droplets on flat‐bottomed 24‐well plates and allowed to solidify for 10–15 min in a 37°C incubator. Following this, each well was supplemented with 500 μL of crypt medium and kept at 37°C in a humidified incubator with 5% CO_2_. The crypt medium was refreshed every 3 days. After 7 days, the organoids were counted and harvested for subsequent processing.

### 4.15 Immunofluorescence and microscopy mouse intestinal organoid

Remove the Mouse intestinal organoid culture medium by aspiration, and carefully wash each well three times with 0.5 mL of PBS, allowing 5 min for each wash. The cells were fixed by adding 0.5 mL of 4% PFA solution and incubating for half an hour at room temperature. Following fixation, the cells were rinsed three times with PBS and gently agitate on a shaker at room temperature for 5 min per wash. To permeabilize the cells, treat them with 0.5 mL of PBS containing 0.1% Triton X‐100 for half an hour at room temperature. After removing the solution, incubate the cells with the primary antibody overnight at 4°C, followed by three washes with 0.1% Triton X‐100 (PBST). After the same washes, the cells were incubated with the secondary antibody (Alexa 488 or 568, Invitrogen, 1:1000) and DAPI for 2 h at room temperature. After this incubation, the cells underwent three additional washes, and an anti‐fluorescence quencher was added drop by drop to each well. All antibodies were used in accordance with the manufacturer's instructions. The specific commercial antibodies employed were anti‐SOX9 (Abways, CY5400, diluted at 1:400) and anti‐Ki67 (Servicebio, GB121141, diluted at 1:300). The imaging was carried out using a Leica TCS‐SP8 confocal microscope and processed using Leica Application Suite X, Adobe Illustrator, Photoshop, and ImageJ software for analysis.

### 4.16 Statistical analysis

All statistical analyses were conducted using GraphPad Prism version 8.0. The data shown are presented as means ± SDs from a minimum of three independent experiments. Statistical significance and sample sizes are indicated in the figures. Differences between groups were assessed using a two‐tailed Student's *t* test unless stated otherwise in the figure legends. A significance level of *p* < 0.05 was considered statistically significant for all analyses. All ROIs in the statistical graphs were 184.52 μm × 184.52 μm.

## AUTHOR CONTRIBUTIONS

Conceptualization, H.C., and T.L.; methodology, T.L., and L.Z.; formal analysis, T.L., and C.F.; investigation, T.L., L.Z., C.F., J.Y., and Y.Y.; data curation, T.L., and Y.Y.; writing‐original draft preparation, T.L.; writing—review and editing, T.L., L.Z., C.F., J.Y., Y.Y., and H.C.; supervision, H.C.; funding acquisition, H.C. All authors have read and agreed to the published version of the manuscript.

## FUNDING INFORMATION

This work was supported by the National Natural Science Foundation of China (92157109) (HC), the National Key Research and Development Program of China (2020YFA0803602), and the 1.3.5 project for disciplines of excellence, West China Hospital, Sichuan University (ZYYC20024) (HC). The funders had no role in the study design, data collection, and analysis, decision to publish, or manuscript preparation.

## CONFLICT OF INTEREST STATEMENT

None declared.

## CONSENT TO PARTICIPATE

Not applicable.

## CONSENT FOR PUBLICATION

All the authors have read and approved the final manuscript.

## Supporting information


Figure S1.

Figure S2.

Figure S3.

Table S1.

Table S2.

Table S3.

## Data Availability

All data generated or analyzed during this study are included in this published article and its supplementary information files. Raw datasets and detailed protocols used during the current study are available from the corresponding author upon reasonable request.GraphPad Prism version 8.0 can be accessed for download at https://www.graphpad.com/. Adobe Photoshop CC 2021 and Adobe Illustrator 2020 are both accessible for download at https://www.adobe.com/products/catalog.html.The RNA‐seq raw data that support the findings of this study have been deposited in the Sequence Read Archive (SRA) under BioProject ID: PRJNA1138061 (https://submit.ncbi.nlm.nih.gov/subs/sra/SUB14607270/overview). For any further inquiries regarding the data, please contact [Haiyang Chen, chenhy82@scu.edu.cn].
