# Supplementary figures and images for: Asparagine prevents intestinal stem cell aging via the autophagy‐lysosomal pathway
Source: Aging Cell. 2024 Nov 25;24(4):e14423. doi: 10.1111/acel.14423 (PMC11984690; doi:10.1111/acel.14423)

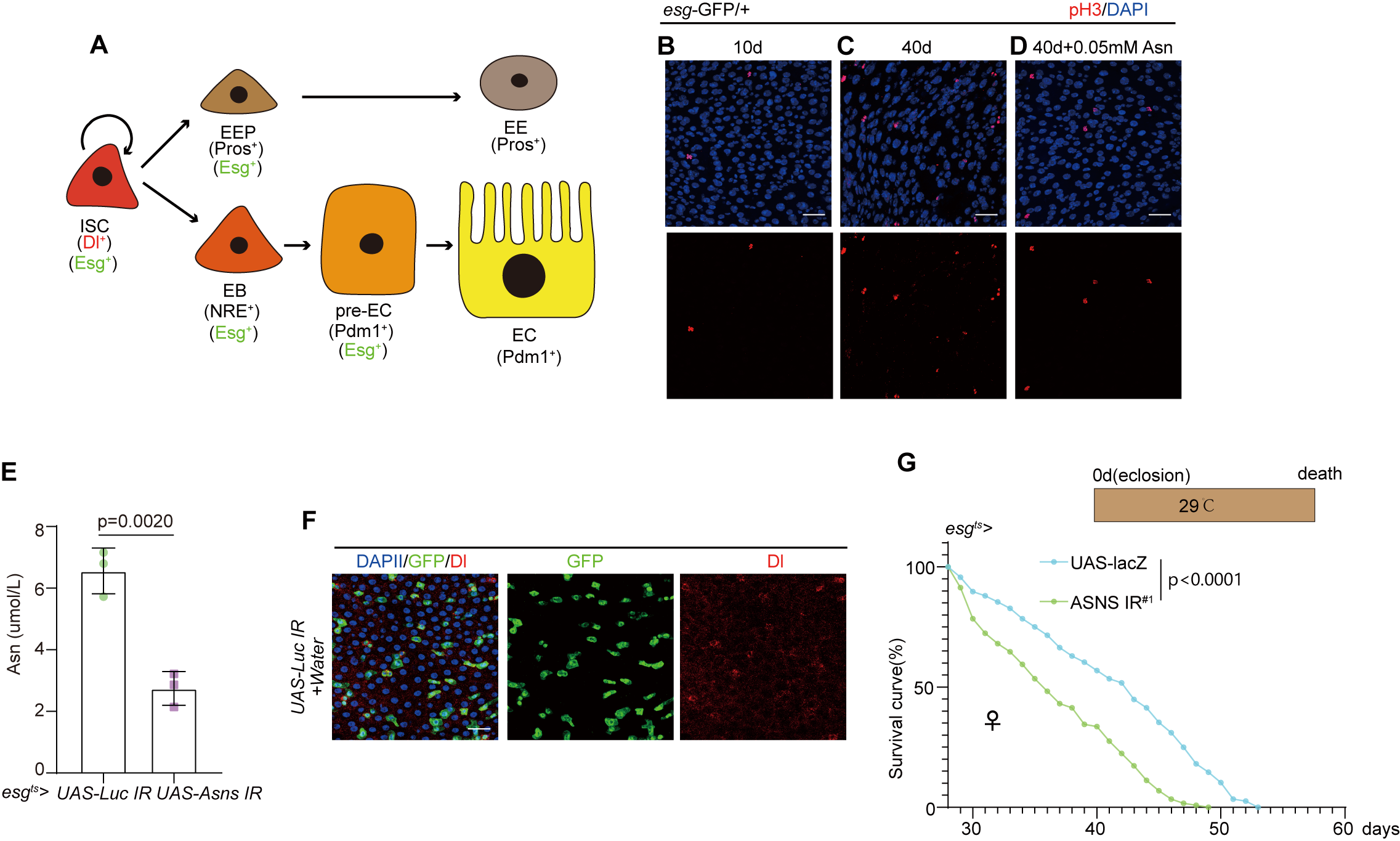

Supplement: Supplementary file 1 — Figure S1. [file ACEL-24-e14423-s002.tif]

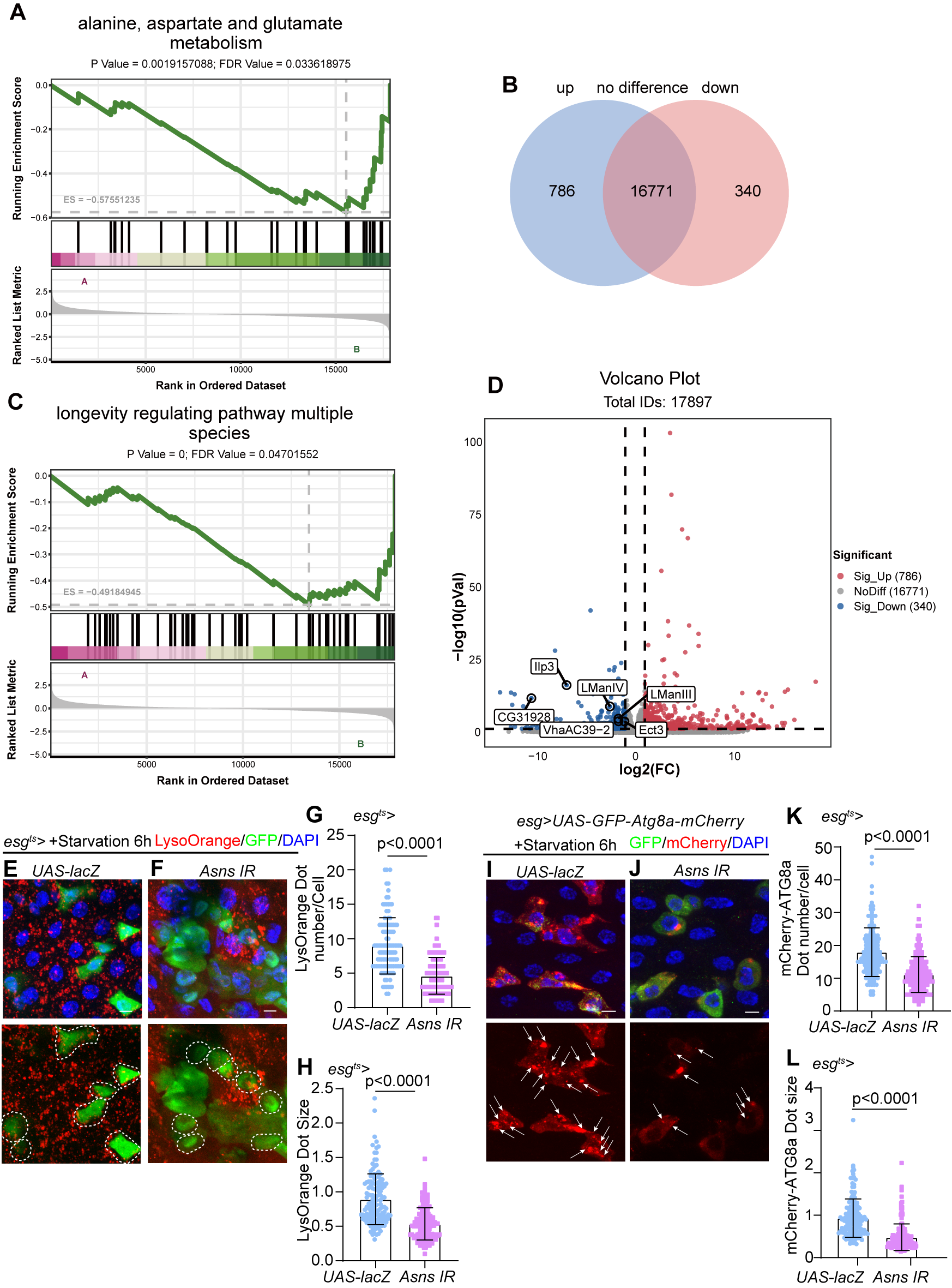

Supplement: Supplementary file 2 — Figure S2. [file ACEL-24-e14423-s004.tif]

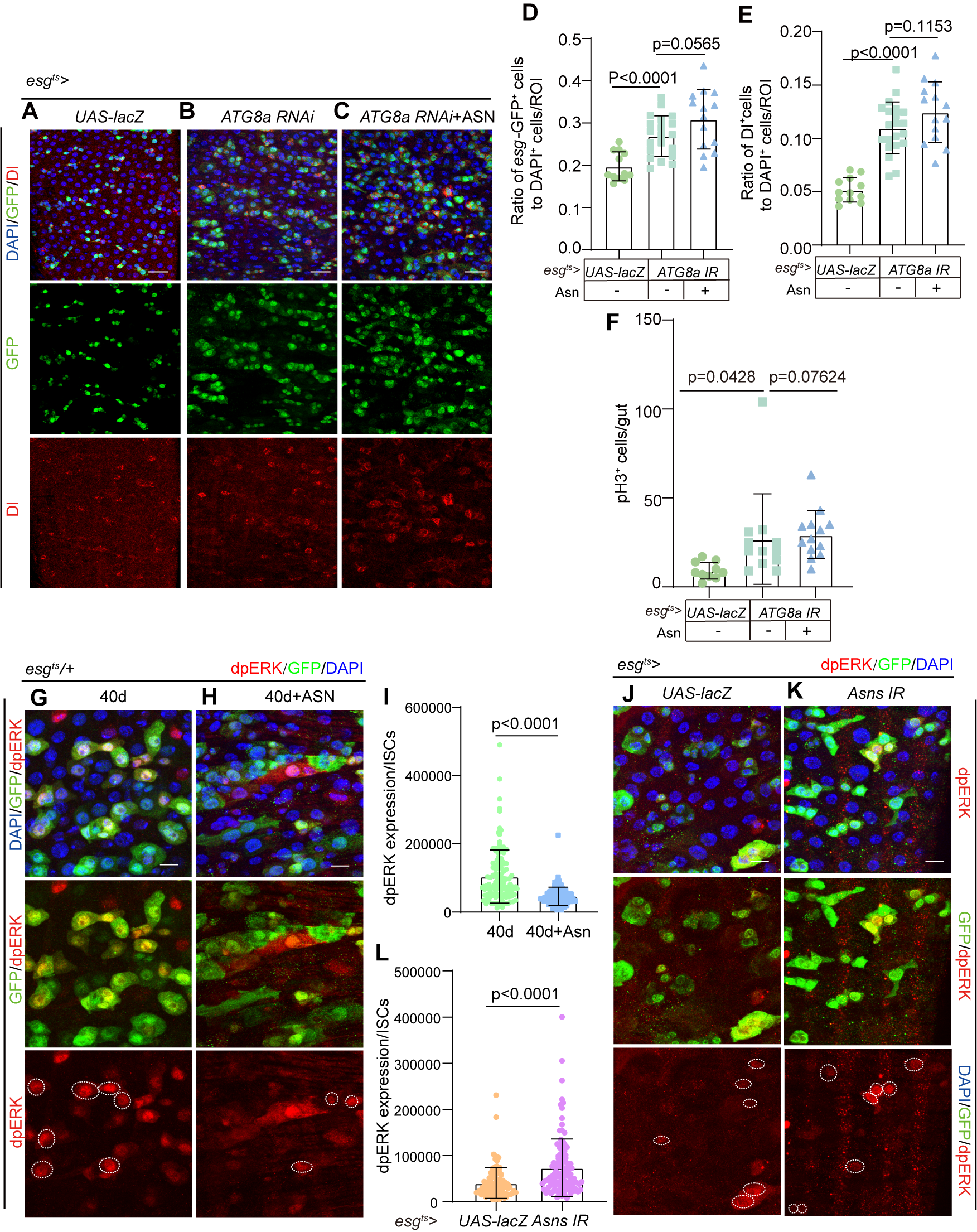

Supplement: Supplementary file 3 — Figure S3. [file ACEL-24-e14423-s006.tif]
